# Determination of the median effective dose of sufentanil combined with remimazolam in inhibiting tracheal intubation response

**DOI:** 10.1038/s41598-025-08907-1

**Published:** 2025-07-02

**Authors:** Lin Feng, Liang Zeng, Xuelei Zhou, Yiping Guo, Xianchun Liu, Longyi Zhang, Ting Zhang, Linji Li, Li Zhao

**Affiliations:** 1 Department of Anesthesiology, The Second Clinical Medical College, North Sichuan Medical College, Beijing Anzhen Nanchong Hospital of Capital Medical University & Nanchong Central Hospital, Nanchong, China; 2 Nanchong Center for Disease Control and Prevention, Nanchong, China

**Keywords:** Sufentanil, Remimazolam, Tracheal intubation, Median effective dose (ED50), Anesthesia induction, Medical research, Drug development, Drug safety

## Abstract

To determine the median effective dose (ED50) and 95% effective dose (ED95) of sufentanil combined with remimazolam for inhibiting the tracheal intubation response in patients undergoing general anesthesia and to evaluate the hemodynamic stability and adverse events associated with this drug combination. This prospective dose-finding study used Dixon’s up-and-down sequential allocation method. A total of 36 patients undergoing general anesthesia surgery between April 2024 and June 2024 were enrolled. Patients were administered remimazolam for induction, followed by sufentanil at an initial dose of 0.4 µg/kg, with subsequent doses adjusted based on the presence or absence of an intubation response. The primary outcome was the ED50 of sufentanil combined with remimazolam, and the secondary outcomes included patient baseline characteristics, hemodynamic parameters, and adverse events. The ED50 and ED95 of sufentanil for inhibiting tracheal intubation response were 0.374 µg/kg (95% CI: 0.342–0.402 µg/kg) and 0.436 µg/kg (95% CI: 0.406–0.586 µg/kg), respectively. Patients with a positive tracheal intubation response had significantly higher heart rates and mean arterial pressures and a higher incidence of hypertension. The ED50 and ED95 of sufentanil combined with remimazolam for inhibiting the tracheal intubation response in patients undergoing general anesthesia were 0.374 and 0.436 µg/kg, respectively. This study provides valuable insights into the dosing of these drugs for effective anesthesia induction and hemodynamic control during tracheal intubation.

## Introduction

Tracheal intubation for general anesthesia is a routine airway preparation for surgical procedures. The tracheal intubation response is the process of stimulating the rich sympathetic nerves of the pharynx and tracheal mucosa to increase catecholamine release during laryngoscope placement and tracheal intubation after the induction of anesthesia. The intense stress caused by the reaction to tracheal intubation can lead to tachycardia and hypertension in a short period of time and even lead to complications such as malignant arrhythmias and cardiovascular accidents^1,2^.

Sufentanil, a potent opioid analgesic, stimulates mu-opioid receptors and is known to inhibit adrenal secretion, increase vagal tone, and suppress metabolism and stress responses. Sufentanil can block afferent signals from laryngeal stimulation during tracheal intubation, thereby reducing the cardiovascular responses to intubation^3,4^. However, inappropriate sufentanil dosing can lead to adverse effects. If the sufentanil dose is too low, it may result in a significant tracheal intubation response, causing severe adverse outcomes. If the dose is too high, the cardiovascular function can be severely suppressed, leading to hypotension after induction^5,6^. Therefore, determining the appropriate sufentanil dose for general anesthesia induction is crucial.

Remimazolam, a novel benzodiazepine sedative, primarily acts on the gamma-aminobutyric acid A receptor^7,8^. Compared to midazolam, remimazolam offers advantages such as rapid onset, fast metabolism, minimal accumulation, less respiratory and circulatory suppression, and faster recovery from anesthesia. Additionally, remimazolam is hydrolyzed by non-specific plasma esterases into an inactive metabolite that does not affect liver and kidney function. Moreover, remimazolam can be rapidly reversed with flumazenil, allowing for more precise anesthetic management^9^. A meta-analysis of 738 patients showed that the sedative effect of remimazolam during general anesthesia was similar to that of propofol, with less impact on hemodynamics^10^. Another randomized controlled trial comparing etomidate and remimazolam for anesthesia induction in cardiac surgery found that remimazolam not only provided stable hemodynamic parameters but also had fewer adverse reactions for anesthesia in cardiac patients^11^. However, there is a scarcity of research on the dose of sufentanil combined with remimazolam for anesthesia induction in adults. Therefore, this study aimed to determine the median effective dose of sufentanil when combined with remimazolam using Dixon’s up-and-down sequential allocation method to provide a reference for clinical application.

## Methods

### Ethical approval

This was an effective dose-finding study based on Dixon’s up-and-down method. The study was approved by the Ethics Committee of Nanchong Central Hospital (2023, trial (61) No.). The study is in the process of registration in the Chinese Clinical Trial Registry (Available at https://www.chictr.org.cn/showproj.html?proj=207603; Registration time: 20/09/2023; Registration Number: ChiCTR2300075949). We confirm that our research is consistent with the Declaration of Helsinki.

### Participants

This study enrolled 36 patients who underwent general anesthesia surgery between April 2024 and June 2024. The inclusion criteria were: age 18–65 years; body mass index (BMI) 19–28 kg/m², and American Society of Anesthesiologists (ASA) physical status I–III. The exclusion criteria were as follows: severe cardiopulmonary diseases; baseline systolic blood pressure > 160 mmHg or heart rate > 100 beats/min; abnormalities in liver or kidney function; history of allergy to study medications; and difficult airway.

### Anesthesia

Patients were required to fast for 8 h and refrain from consuming liquids for 6 h prior to surgery. Upon arrival in the operating theater, venous access was secured for the administration of a compound sodium chloride solution, and oxygen was supplied at a rate of 3 L/min through a face mask. Vital signs, including electrocardiography (ECG), pulse oximetry (SpO2), heart rate (HR), blood pressure, and bispectral index (BIS), were continuously monitored. HR and blood pressure were measured three times consecutively after the patient entered the operating theatre for a 10-minute rest and averaged as the baseline values. Anesthesia induction was achieved through a gradual intravenous infusion of 0.25 mg/kg of remimazolam. After BIS ≤ 60 and MOAA/S (Modified Observer’s Assessment of Alertness/Sedation) ≤ 1, sufentanil 0.4 µg/kg and cisatracurium 0.2 mg/kg were administered intravenously. Neuromuscular blockade was assessed using a train-of-four (TOF) peripheral nerve stimulator. Tracheal intubation was performed 5 min after sufentanil administration and only when TOF counts reached 0/4. Tracheal intubation was performed using a video laryngoscope by an anesthesiologist with over ten years of experience, and the tube was lubricated with lidocaine hydrochloride jelly. The standardized tube sizes were as follows: 7.5 mm ID for men and 7.0 mm ID for women, with an increase or decrease of 0.5–1.0 mm for short/thin or tall/obese patients. After successful intubation, mechanical ventilation was initiated. The initial dose of sufentanil was set at 0.4 µg/kg for the first patient, with subsequent doses adjusted based on the presence or absence of an intubation response. In this study, positive tracheal intubation reaction was defined as an increase in mean arterial pressure (MAP) or HR of more than 20% from baseline or BIS > 60 during intubation and within 2 min after intubationduring intubation and within 2 min after intubation^12^. If no intubation response occurred, the sufentanil dose was decreased by 0.05 µg/kg for the next patient. If the intubation response was observed, the sufentanil dose was increased by 0.05 µg/kg for the next patient. If an intubation response occurs during intubation, additional remimazolam or sufentanil should be administered as needed. Throughout the surgery, sevoflurane was used to maintain the depth of anesthesia, with intermittent boluses of sufentanil and cisatracurium given to maintain analgesia and muscle relaxation. After surgery, the endotracheal tube was removed, and the patient was transferred to the Post-Anesthesia Care Unit.

### Determination of ED50

The ED50 of sufentanil for inhibiting tracheal intubation response was determined using the up-and-down sequential allocation method. The initial dose of sufentanil was set at 0.4 µg/kg, with a dose gradient of 0.05 µg/kg. Sample size calculations were based on the principles of the sequential allocation method, and the ED50 was calculated by completing the study when seven crossings between successes and failures were reached^13,14^.

### Study outcomes

The primary outcome was the ED50 of sufentanil combined with remimazolam for inhibiting the tracheal intubation response. The secondary outcomes included the following: patient baseline characteristics (age, sex, height, weight, BMI, ASA classification, etc.). Hemodynamic parameters were recorded at T1(baseline), T2 (1 min after induction), and T3 (2 min after intubation). Adverse events during anesthesia induction were recorded: increased blood pressure, decreased blood pressure (30% above baseline), bradycardia and tachycardia (heart rate < 50 or > 100 beats/min), hypoxemia (SpO2 < 90%), body movement, and injection pain.

### Statistical analysis

The sample size for this study was based on the sequential allocation method. According to this principle, the ED50 can be accurately calculated when the sample size reaches seven crossings^13,14^. ED50 and ED95 were estimated using probabilistic regression analysis in SPSS. The positive and negative groups were divided according to the intubation response. Normally distributed data are expressed as mean ± standard deviation and analyzed using the Independent Samples t-test. Non-normally distributed data were expressed as median and interquartile range and analyzed using the Kruskal-Wallis H test. Qualitative data were expressed as frequencies and percentages and analyzed using the chi-square test. Statistical significance was set at *p* < 0.05.

## Results

**Fig. 1:**
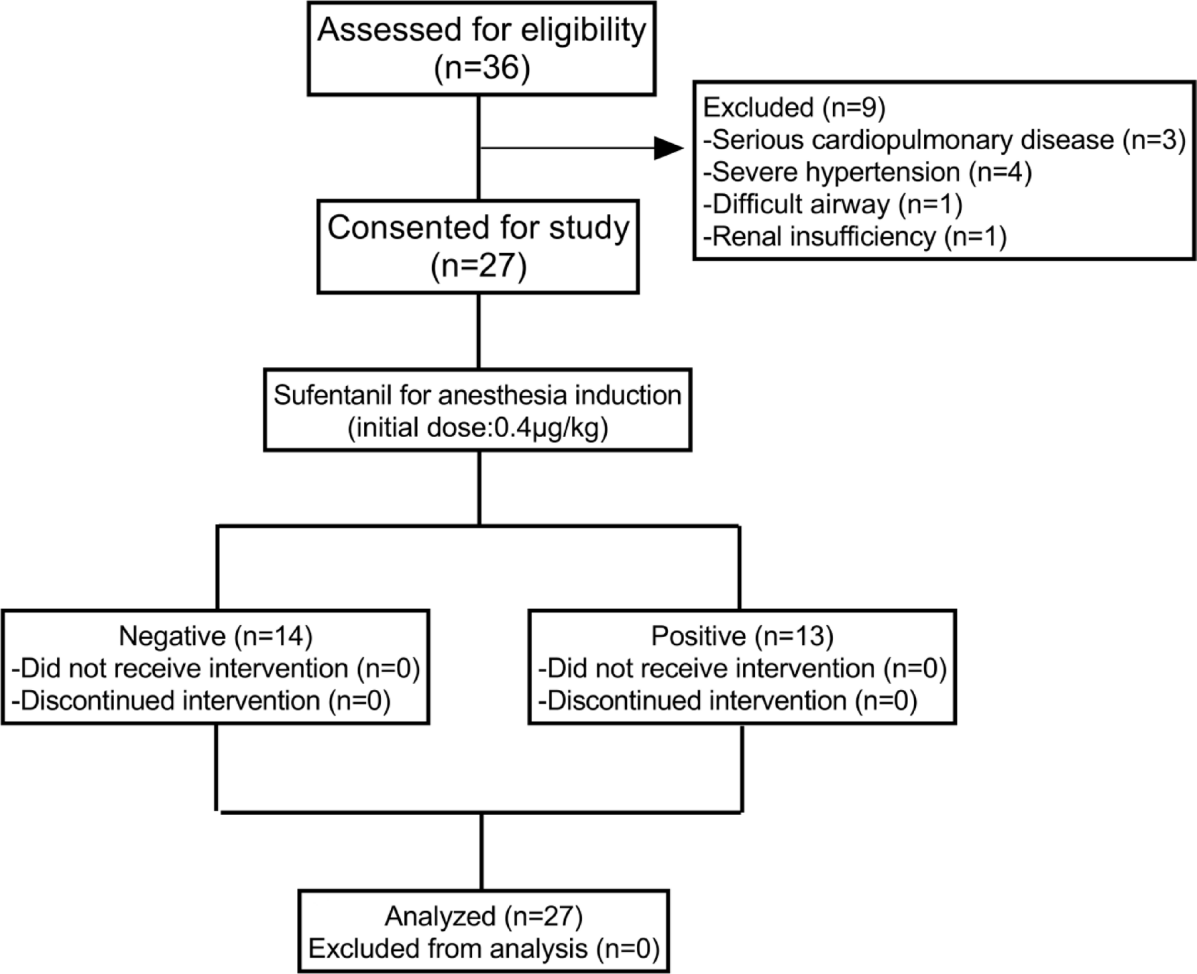
Study flow chart.

The flow chart for this study is shown in Fig. 1. We will assess 36 patients for eligibility between April and June 2024. Nine patients were excluded: three with severe cardiac disease, four with severe hypertension, one with renal insufficiency, and one with difficult airway. A total of 27 patients were included in the final analysis, of whom 14 had a negative tracheal intubation reaction and 13 had a positive reaction. The basic profiles of the patients are shown in Table 1.

**Table 1:** Characteristics of the included studies.

Item	Negative (*n* = 14)	Positive (*n* = 13)	*P*
Sex (M/F)	5/9	5/8	1
Age (years)	48.6 ± 7.7	46.6 ± 9.9	0.561
Weight (kg)	63.0 ± 7.4	64.8 ± 8.8	0.555
BMI (kg/m^2^)	24.5 ± 2.1	24.8 ± 2.5	0.805
ASA (I/II)	1/13	0/13	1
Hypertension	2 (14.3%)	2 (15.3%)	0.97
Diabetes	0	1 (7.7%)	0.97
Smoking status (within 1 month)	3 (21.4%)	2 (15.4%)	1

Data are presented as mean ± SD or number (percentage of patients).

*BMI* body mass index, *ASA* American Society of Anesthesiologists. Smoking Status.

**Fig. 2:**
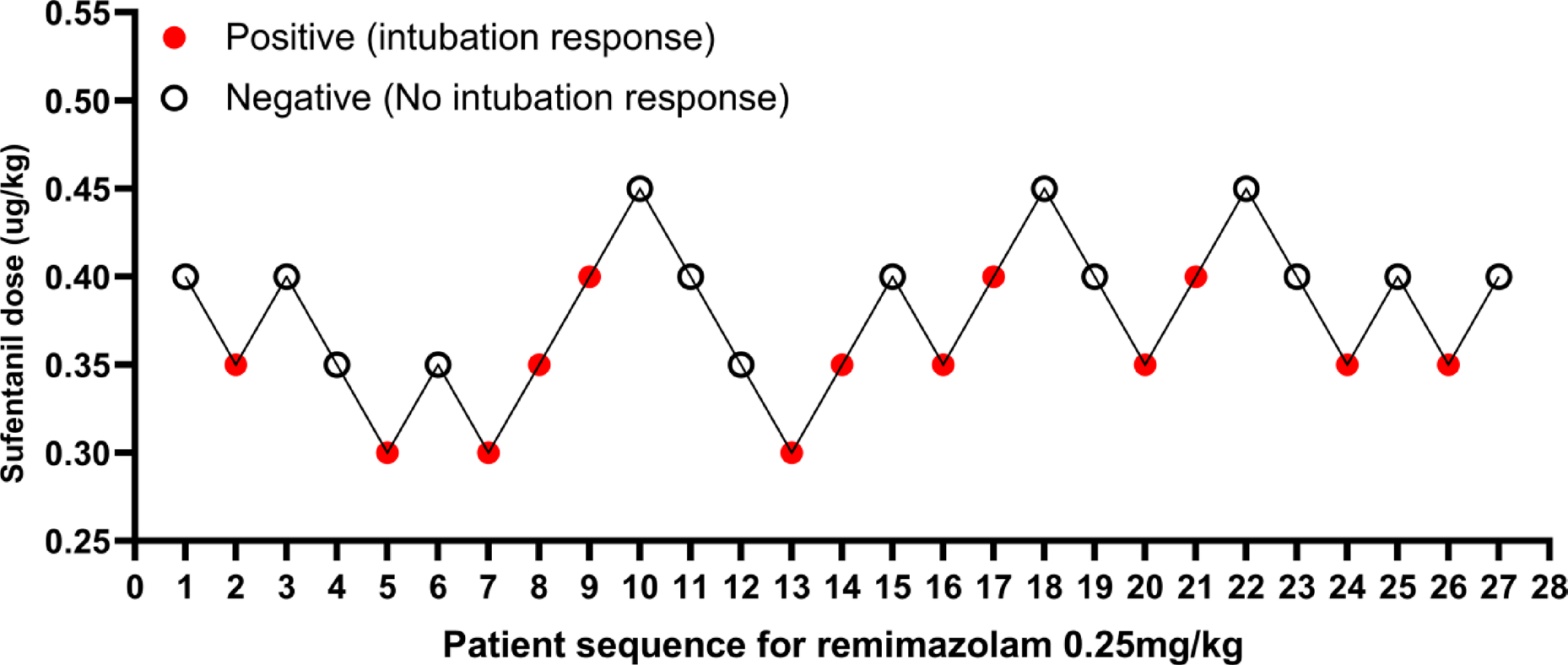
Patient up-down chart.

**Fig. 3:**
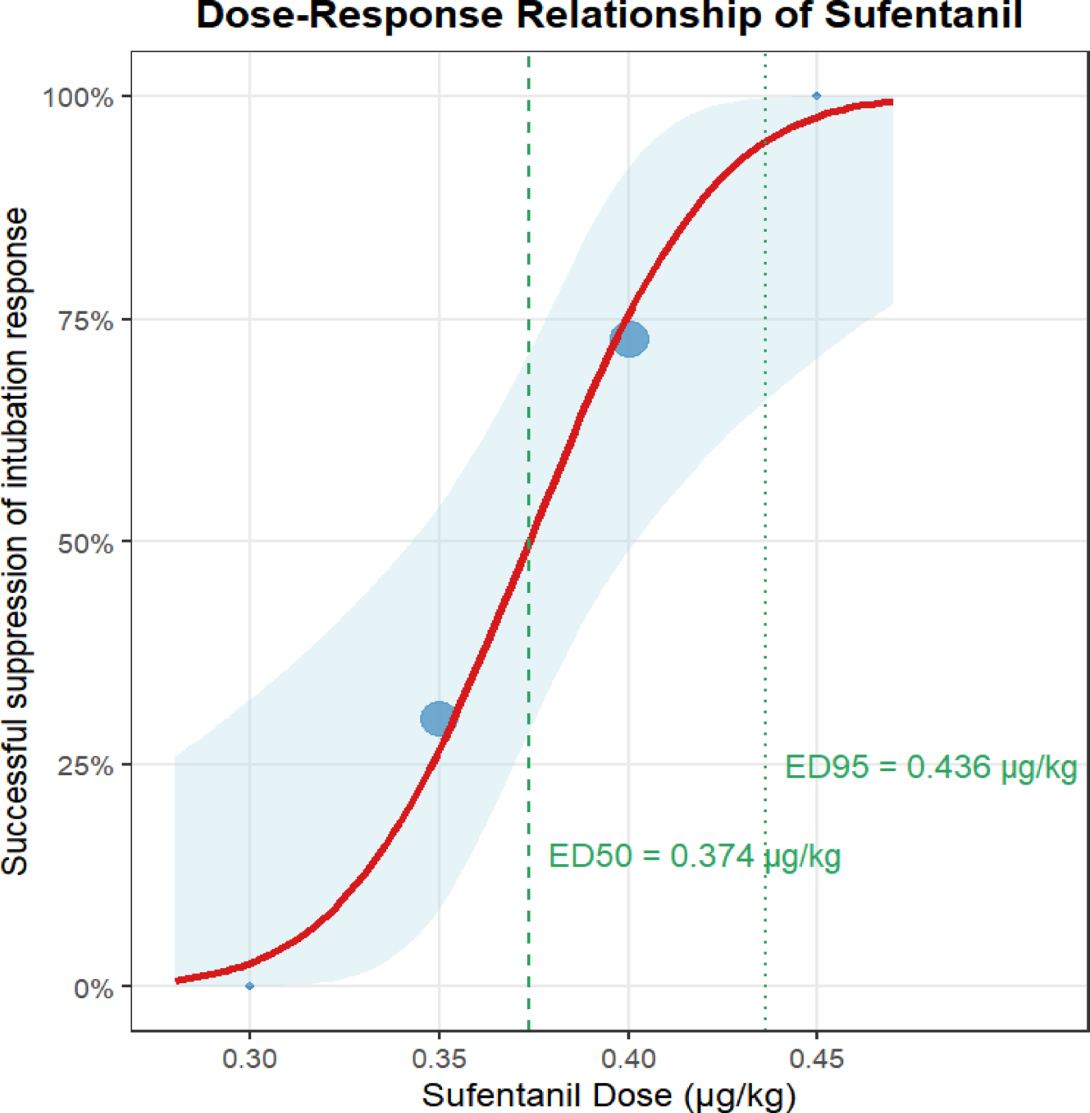
Dose-response relationship of sufentanil.

Figure 2 shows the up-and-down sequential response to the induction of intubation with sufentanil in 36 patients. The numbers of positive and negative tracheal intubations induced by different doses of sufentanil are shown in Table 2. At 0.45 µg/kg sufentanil induction, the tracheal intubation response was negative in all patients. Tracheal intubation response was positive in all patients at a dose of 0.30 µg/kg sufentanil. Our study ended the trial after seven positive-negative crossovers. Figure 3 shows the dose-response relationship of sufentanil.

Table 3 shows that the ED50 and 95% confidence intervals (CI) of sufentanil combined with remimazolam for inhibiting tracheal intubation response were 0.374 (0.342–0.402)µg/kg and 0.436 (0.406–0.586) µg/kg, respectively.

**Table 2:** Number of positive and negative intubations induced by different doses of sufentanil.

Sufentanil dose	Negative (number)	Positive (number)	Sample size (number)
0.30 µg/kg	0	3	3
0.35 µg/kg	3	7	10
0.40 µg/kg	8	3	11
0.45 µg/kg	3	0	3

**Table 3:** ED50 and ED95 and their 95% CI.

	Sufentanil for induction	95%CI for sufentanil
ED50 (µg/kg)	0.374	0.342–0.402
ED95 (µg/kg)	0.436	0.406–0.586

**Fig. 4:**
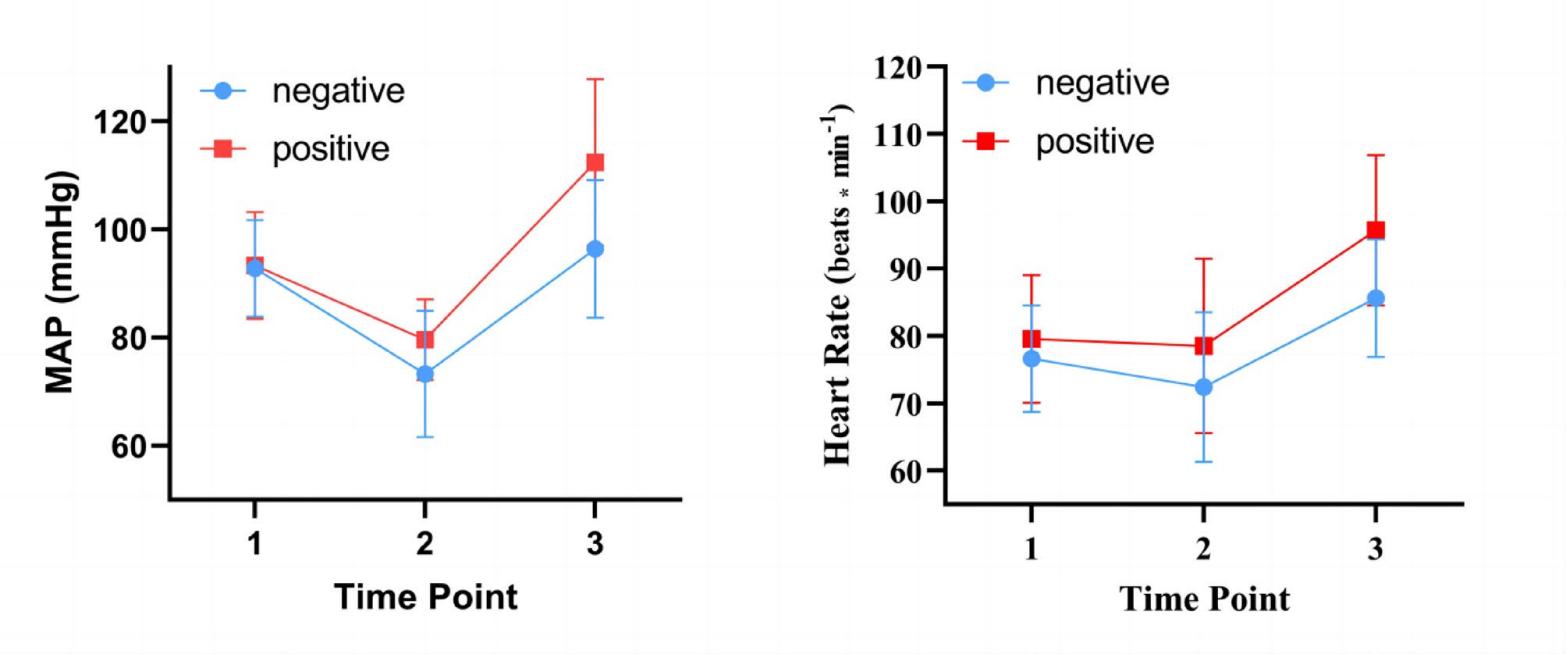
Shows the data results for HR and MAP. *MAP* mean arterial pressure, *HR* heart rate, *T1* before induction, *T2* 1 min after induction, *T3* at a maximum of 2 min after tracheal intubation.

**Table 4:** Hemodynamic data.

Item	Time	Negative (*n* = 14)	Positive (*n* = 13)
Heart rate (beats/min)	T1	76.6 ± 7.9	79.5 ± 9.5
	T2	72.4 ± 11.1	78.5 ± 13.0
	T3	85.6 ± 8.7	95.7 ± 11.7
Mean arterial pressure (mmHg)	T1	92.8 ± 8.9	93.4 ± 9.9
	T2	73.3 ± 11.7	79.6 ± 7.5
	T3	96.4 ± 12.8	112.5 ± 15.4

Data are presented as mean ± SD. T1: before induction; T2:1 min after induction; T3: at a maximum of 2 min after tracheal intubation. Data were analyzed using the Iidependent samples t-test.

The hemodynamic data of the patients are shown in Table 4; Fig. 4. The heart rate results were as follows: In the intra-group comparison between the positive and negative groups, HR was significantly lower at T2 than at T1 and T3 (*p* < 0.05). In the between-group comparison, HR was significantly higher in the positive group than in the negative group at T3 (*p* = 0.015).

The results for MAP were as follows: MAP was significantly lower in the negative group at T2 than at T1 and T3 (*p* < 0.05). In the positive group, MAP was significantly lower at T2 than at T1 and T3 (*P* < 0.05) and significantly higher at T3 than at T1. In the between-group comparison, HR was significantly higher in the positive group than in the negative group at T3 (*p* = 0.007).

The incidence of adverse events in this study is presented in Table 5. The incidence of hypertension was significantly higher in the positive group (*p* = 0.038). No statistical difference was observed in the comparison of other adverse events. In addition, no patient experienced hypoxemia or injection pain.

**Table 5:** The incidence of adverse events.

Adverse events	Negative (*n* = 14)	Positive (*n* = 13)	*P*
Hypertension, n(%)	0 (0%)	5 (38.5%)	0.038
Hypotension, n(%)	4 (28.6%)	1(7.7%)	0.468
Bradycardia, n(%)	2 (14.3%)	0	0.496
Tachycardia, n(%)	0 (0%)	2(15.4%)	0.43
Hypoxaemia, n(%)	0 (0%)	0 (0%)	/
Injection pain, n(%)	0 (0%)	0 (0%)	/
Body movement, n(%)	0 (0%)	1 (7.7%)	0.97

Data are presented as mean ± SD or number (percentage of patients).

## Discussion

In this study, we determined the median effective dose (ED50) and 95% effective dose (ED95) of sufentanil combined with remimazolam for inhibiting the tracheal intubation response using the up-and-down sequential allocation method. The results indicated that the ED50 and ED95 of sufentanil were 0.374 µg/kg (95% CI: 0.342–0.402 µg/kg) and 0.436 µg/kg (95% CI: 0.406–0.586 µg/kg), respectively.

As a new type of benzodiazepine sedative, remimazolam has the advantages of a fast onset of action, rapid metabolism, low accumulation, minimal respiratory and circulatory depression, and precise anesthetic effect^15,16^. zhao et al.‘s study of 1147 patients with painless endoscopy in a meta-analysis found that remimazolam significantly reduced the incidence of adverse events, such as injection pain, hypotension, and respiratory depression, compared to propofol^17^. chen et al.‘s study found that induction of anesthesia with remimazolam in combination with sufentanil had a lower incidence of cardiovascular and respiratory adverse events compared to propofol^18^. Because of the stable effect of remimazolam combined with sufentanil during anesthesia induction, this study further explored their dosage. The ED95 of remimazolam for induction of anesthesia in hysteroscopic patients was 0.254 mg/kg in the study by Huang et al. Therefore, we chose 0.25 mg/kg of remimazolam as the induction dose of anaesthesia for this study^19^.

The Dixon sequential method is commonly used clinically for anesthetic drug dose-effect relationship studies and is a simple and rapid method for ED50 determination. The Dixon up-and-down sequential method starts from the best presumptive dose of the given ED50, and each subject is based on the presence or absence of responsiveness of the previous subject to increase or decrease the level of the next dose, which generally requires a sample size of seven crossovers later^20^. The present study required intubation 5 min after sufentanil administration, consistent with the timing of its peak effector compartment concentration^21^, thereby improving the accuracy of the ED50 estimation.

The hemodynamic findings of this study emphasize the clinical significance of effectively controlling tracheal intubation reactions. Our findings showed that the maximum values of both HR and MAP were significantly higher in the positive group than in the control group within 2 min after tracheal intubation. In addition, the highest MAP value in the positive group was significantly higher than the baseline value within 2 min of tracheal intubation, along with a significantly higher incidence of hypertension. These findings emphasize the need for clinical strategies to reduce intubation reactions, which are essential for patient health and to avoid complications that may arise from hemodynamic fluctuations. Smoking history was balanced between groups, but the small subgroup precluded definitive analysis of its impact. future larger studies should Future larger studies should stratify dosing by smoking status, particularly for heavy active smokers, to refine hemodynamic optimization. In this study, hypotension was defined as a > 30% decrease in the MAP from the baseline. Notably, no patient exhibited MAP values below 60 mmHg (the critical threshold for organ perfusion), suggesting that the observed MAP reductions may not have reached clinically critical levels and that the relative blood pressure reductions primarily reflected the dose-dependent hemodynamic effects of sufentanil.

The findings of our study contribute to the clinical understanding of the optimal sufentanil dose when combined with remimazolam for the induction of general anesthesia. The ED50 and ED95 values obtained provide a scientific basis for the administration of these drugs in a manner that balances efficacy and safety. The significance of these results lies in their potential to guide clinical practice towards minimizing hemodynamic responses during tracheal intubation, thereby reducing the risk of adverse outcomes. Notably, lidocaine hydrochloride jelly was applied to the endotracheal tube as part of the standardized intubation protocol. Although this practice may have contributed to suppressing laryngeal reflexes and reducing nociceptive stimuli, it was consistently applied across all participants. Thus, the calculated ED50 and ED95 values reflect the effective dose of sufentanil in this clinical scenario, where adjunctive local anesthesia is routinely used. However, future studies should further investigate the interaction between topical lidocaine and opioid requirements during intubation.

This study has some limitations. First, the sample size calculated based on Dixon’s up-and-down sequential allocation method can satisfy the calculation of ED50; however, it may be insufficient for evaluating secondary outcome indicators. Second, we did not observe the occurrence of sufentanil-related postoperative adverse effects, such as postoperative nausea and vomiting and postoperative delirium. Third, while we collected smoking history, the small subgroup of recent smokers (*n* = 5) limited analysis of its influence on hemodynamic responses. Future research should explore the dose-response relationship in a more diverse patient population, including those with different comorbidities and in various surgical settings.

## Conclusion

The median effective dose (ED50) and 95% effective dose (ED95) of sufentanil combined with remimazolam for inhibiting the tracheal intubation response in patients undergoing general anesthesia were determined to be 0.374 µg/kg and 0.436 µg/kg, respectively.

## Data Availability

The datasets generated and analyzed during the current study are available from the corresponding author upon reasonable request.
